# Whole Genome Sequence Analysis of a Prototype Strain of the Novel Putative Rotavirus Species L

**DOI:** 10.3390/v14030462

**Published:** 2022-02-24

**Authors:** Reimar Johne, Katja Schilling-Loeffler, Rainer G. Ulrich, Simon H. Tausch

**Affiliations:** 1 Department of Biological Safety, German Federal Institute for Risk Assessment, 10589 Berlin, Germany; katja.schilling-loeffler@bfr.bund.de (K.S.-L.); simon.tausch@bfr.bund.de (S.H.T.); 2 Institute of Novel and Emerging Infectious Diseases, Friedrich-Loeffler-Institut, Federal Research Institute for Animal Health, 17493 Greifswald-Insel Riems, Germany; rainer.ulrich@fli.de; 3 German Centre for Infection Research (DZIF), 17493 Greifswald-Insel Riems, Germany

**Keywords:** rotavirus, genome sequence, taxonomy, phylogeny, virus species

## Abstract

Rotaviruses infect humans and animals and are a main cause of diarrhea. They are non-enveloped viruses with a genome of 11 double-stranded RNA segments. Based on genome analysis and amino acid sequence identities of the capsid protein VP6, the rotavirus species A to J (RVA-RVJ) have been defined so far. In addition, rotaviruses putatively assigned to the novel rotavirus species K (RVK) and L (RVL) have been recently identified in common shrews (*Sorex araneus*), based on partial genome sequences. Here, the complete genome sequence of strain KS14/0241, a prototype strain of RVL, is presented. The deduced amino acid sequence for VP6 of this strain shows only up to 47% identity to that of RVA to RVJ reference strains. Phylogenetic analyses indicate a clustering separated from the established rotavirus species for all 11 genome segments of RVL, with the closest relationship to RVH and RVJ within the phylogenetic RVB-like clade. The non-coding genome segment termini of RVL showed conserved sequences at the 5′-end (positive-sense RNA strand), which are common to all rotaviruses, and those conserved among the RVB-like clade at the 3′-end. The results are consistent with a classification of the virus into a novel rotavirus species L.

## 1. Introduction

Rotaviruses are a main cause of acute diarrhea in children and young animals. It has been estimated that 128,500 deaths among children younger than 5 years worldwide were attributed to rotavirus infections in 2016 [1]. Rotaviruses have also been described as an important cause of diarrhea in domestic mammals [2,3] and poultry [4,5]. In addition, they have been detected in several wild animal species including wild boars, bats, rats and shrews [6,7,8,9]. As the zoonotic transmission of some rotaviruses has been described, animal rotaviruses may also play a role for human rotavirus epidemiology [10]. 

Rotaviruses are non-enveloped particles with a genome consisting of 11 segments of double-stranded RNA [11]. Each of the genome segments either encodes one of the structural proteins VP1—VP4 and VP6—VP7, or one of the non-structural proteins NSP1—NSP5. In some rotaviruses, an additional open reading frame for NSP6 is present on the NSP5-encoding segment [12]. The coding regions are flanked by non-coding regions (NCRs) at the 5′- and 3′-ends of the genome segments, which contain nucleotide positions highly conserved among rotaviruses of the same species [13]. 

The different rotavirus species are all grouped into the genus *Rotavirus* within the family *Reoviridae*. Until now, 9 rotavirus (RV) species designated as RVA to RVD and RVF to RVJ have been officially adopted by the International Committee on Taxonomy of Viruses (ICTV) [14]. Serological cross-reactivity with antibodies against the capsid protein VP6 and distinct host ranges of the viruses can be used as criteria to distinguish the rotavirus species [13]. However, a cut-off value of 53% identity of the VP6 amino acid sequence can be more easily used to differentiate members of different rotavirus species [15]. Based on phylogenetic analyses [16] and sequence comparisons of the VP1 [17], two major clades of rotaviruses have been identified, which are the RVA-like clade (containing RVA, RVC, RVD, RVF) and the RVB-like clade (containing RVB, RVG, RVH). 

Recently, rotaviruses with low sequence identities to other mammalian and avian rotaviruses have been detected in common shrews (*Sorex ananeus*) from Germany [9]. Some of these virus strains could be classified into a divergent evolutionary branch within RVA [18]. Other strains were putatively grouped into a proposed species designated as RVK, which is most closely related to RVC, or another proposed species designated as RVL, which is most closely related to RVH [9]. However, these classifications are preliminary as only partial genome sequences were available for these rotaviruses so far.

Here, the complete genome of a prototype strain of RVL from a common shrew was sequenced. Sequence analyses of all genome segments indicated only low identities to the other rotavirus species. Together with the results of the phylogenetic analysis, the data support the grouping into a new species RVL. Based on phylogenetic clustering and conserved sequences at the 3′-termini of the genome segments, RVL has to be considered as a new member of the RVB-like clade of rotaviruses.

## 2. Materials and Methods

### 2.1. Screening of Samples for RVL-RNA

The collection of 46 wild common shrews from Germany was described previously [9]. Collection of shrews was based on snap trapping, or the shrews were found dead in live traps. The animals were initially stored frozen and intestinal contents (~0.2 g) were collected later from thawed animals; the intestinal contents were again frozen at −20 °C until further analysis. Thereafter, they were diluted 1:5 in phosphate-buffered saline, and 100 μL of the solution were subsequently subjected to nucleic acid extraction using the NucliSENS^®^ easyMAG system (BioMerieux, Marcy I’Etoile, France) according to the manufacturer’s instructions. The samples were screened by reverse transcription (RT-)PCR for the presence of RVL-RNA using primers ShrewRVL-s (5′-TGA TCT GCT TGC TAT GAA ATA TGA-3′) and ShrewRVL-as (5′-ATC TAG TTG GAT GTT ATC AAT CAT-3′), which have been designed based on available partial RVL sequences. The expected amplicon is a 291 base pair (bp) fragment of the VP1-encoding genome segment. The One-Step RT-PCR kit (QIAGEN, Hilden, Germany) was used with a 2720 thermal cycler (Applied Biosystems, Waltham, MA, USA). The thermal profile comprised 42 °C for 30 min and 95 °C for 15 min, followed by 40 cycles at 94 °C for 30 s, 56 °C for 30 s, and 74 °C for 40 s, with a final extension at 74 °C for 5 min. PCR products were separated by electrophoresis on ethidium bromide-stained agarose gels. 

### 2.2. Whole Genome Sequencing

Extracted nucleic acids from the selected RVL-positive sample (see Section 3.1) were treated with DNase as previously described [9] and used for library preparation with the KAPA RNA HyperPrep Kit (Roche Diagnostic, Mannheim, Germany) and the KAPA Unique Dual-Indexed Adapter Kit for Illumina^®^ platforms (Roche Diagnostic) as per manufacturer’s instructions. During library preparation, RNA was fragmented at 85 °C for 6 min. The input nucleic acid concentration was low (<1 ng), therefore the adapter stock for ligation was diluted to a concentration of 0.15 µM, and the library was amplified in 24 cycles using the KAPA Hifi HotStart Ready Mix 2× (Roche Diagnostics) after ligation. The resulting library was purified using KAPA Pure Beads (Roche Diagnostics) and size distribution monitored on the Fragment Analyzer 5200 (Agilent Technologies, Santa Clara, CA, USA) using the Agilent DNF-474 HS NGS Kit (Agilent Technologies). The final library was pooled with 126 additional libraries and sequenced with 2 × 150 cycles on the NextSeq 500 Sequencer (Illumina, San Diego, CA, USA) using the NextSeq 500/550 Mid Output Kit v2.5 (Illumina).

The generated raw sequences were trimmed using the fastp module [19] of the AQUAMIS pipeline [20]. Remaining reads were subjected to assembly using SPAdes [21]. All resulting contigs were screened for sequence similarities with rotavirus sequences using BLASTX [22] against all rotavirus sequences available from the NCBI Protein database [23] (https://www.ncbi.nlm.nih.gov/protein/, accessed on 20 October 2021).

Missing terminal sequences of the genome segments were amplified by rapid amplification of cDNA ends (RACE) using the 5′ RACE System Kit (Invitrogen GmbH, Karlsruhe, Germany), with specific primers delineated from next generation sequencing (NGS)-derived sequences. Products with the expected lengths were purified using the QIAquick DNA Purification Kit (QIAGEN) and subjected to dideoxy chain termination sequencing by a commercial supplier (Eurofins, Ebersberg, Germany). 

### 2.3. Sequence Analysis

The SeqBuilder module of the DNASTAR software package (Lasergene, Madison, WI, USA) was used for assembly of NGS contigs together with RACE-derived terminal sequences and for translation of open reading frames (ORFs) to amino acid sequences. The complete nucleotide sequences of the genome segments were submitted to the GenBank database with accession numbers OM101015-OM101025.

The genome sequence of the RVL strain was compared with that of reference strains for the other rotavirus species RVA, RVB, RVC, RVD, RVF, RVG, RVH, RVI and RVJ. As RVE is not an official rotavirus species and only partial sequences are available for RVK, both species have been excluded from further analysis. The distinct strain designations and the GenBank accession numbers of the used sequences are presented in Supplementary Data S1. Alignments of deduced amino acid sequences and calculation of amino acid sequence distances and identities were done using the MegAlign Pro module of the DNASTAR software package (Lasergene) by applying the ClustalW method with default parameters in alignments. Phylogenetic analyses were performed using MEGA X version 10.1.7 [24]. Briefly, the deduced amino acid sequences were aligned by the ClustalW method with default parameters using MEGA X. Phylogenetic reconstructions were thereafter performed using MEGA X by the Neighbor-joining method with the following parameters: 1000 bootstrap replications, Poisson model for amino acid substitutions, uniform rates among sites, pairwise deletion of gaps or missing sequences. The resulting trees were manually labeled and formatted using Microsoft Powerpoint. 

## 3. Results

### 3.1. RVL Screening and Sample Selection for NGS

A screening of intestinal content samples of 46 wild common shrews using a newly developed RVL-specific RT-PCR resulted in 5 positive samples. Of these, sample KS14/0241 showed the strongest band in the stained agarose gel and was, therefore, selected for further analysis by NGS. This sample originated from a female common shrew with a body weight of 6 g, which was trapped near Stuttgart, south-west Germany, in June 2013. 

### 3.2. RVL Genome Sequencing

By NGS, a total of 3,097,640 paired reads were generated, which resulted in a final set of 2,577,304 paired reads after trimming. The assembly generated 879 contigs, out of which 11 contigs with similarities to already available partial RVL sequences were selected. These contigs had a read coverage between 323 and 493 and represented nearly the whole RVL genome, including all 3′-terminal and most 5′-terminal sequences of the genome segments. Short missing 5′-terminal sequences of genome segments encoding VP3, VP7, NSP1 and NSP5 were sequenced using RACE protocols followed by Sanger sequencing. In addition, the 3′-terminal coding region for NSP3, which contains an unusual poly-proline-encoding region, was amplified by RT-PCR and subjected to Sanger sequencing, which confirmed the respective sequence derived from NGS. 

### 3.3. RVL Genome Organization and Identities with Other Rotavirus Species

The complete RVL genome consists of 18,300 nucleotides (Supplementary Data S2). The 11 genome segments have a coding capacity for proteins with similarities to VP1–VP4, VP6–VP7 and NSP1–NSP5 of other rotaviruses. The VP4 and NSP3 sequences are slightly longer than those of the other rotaviruses (Supplementary Data S2). In VP4, this is due to several short insertions scattered among the whole sequence, most of them with similarities to insertions present in other rotavirus species (Supplementary Data S3). In contrast, the RVL-NSP3 sequence contains one unique insertion in the central part of the sequence and additional amino acid residues at the C-terminus, including a stretch of 8 consecutive proline residues (Supplementary Data S4). Compared to reference strains of other rotavirus species, the encoded proteins of RVL showed amino acid sequence identities ranging from 3% (to NSP4 of RVF) to 64% (to VP1 of RVJ), with generally the highest identities to RVJ and RVH. VP6 of RVL shows a maximum amino acid sequence identity of 47% to that of RVH and RVJ (Table 1).

### 3.4. Analysis of NCRs at the Genome Segment Termini

Comparisons of the NCRs of the RVL genome segments identified conserved nucleotide residues (Table 2). These include a strictly conserved GGC sequence at the 5′-termini (positive-sense RNA strand) and a strictly conserved UAGACCC sequence at the 3′-termini of all RVL segment sequences. A comparison of the NCRs shows the general consensus sequence GGN^A^/_U_ at the 5′-terminus of RVL and all other rotavirus species. In contrast, the last four 3′-terminal nucleotides of RVL have the consensus sequence ACCC, common with the RVB-like clade (RVB, RVG, RVH, RVI, RVJ), whereas the RVA-like clade (RVA, RVD, RVF) shows the sequence GACC, and RVC shows GGCU at the corresponding position (Table 2). 

### 3.5. Phylogenetic Analysis

**Figure 1:**
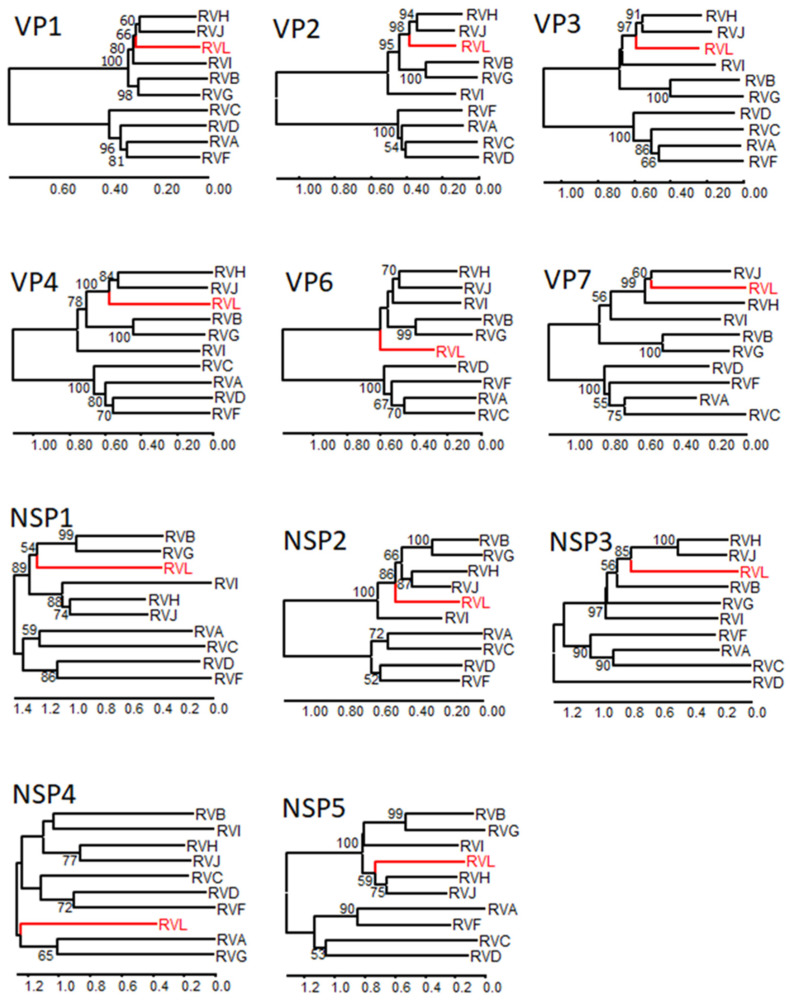
Phylogenetic relationship of RVL with other rotavirus species. The complete deduced amino acid sequences of the encoded proteins of RVL were compared with those of reference strains of the other rotavirus species (as specified in Supplementary Data S1) by the Neighbor-joining method using MEGA X. RVL is marked in red and bootstrap values > 50% are shown. Scaled in amino acid substitutions per site.

As the nucleotide sequences of the different rotavirus species genomes are highly divergent, phylogenetic analyses were performed with the more conserved deduced amino acid sequences. Phylogenetic trees generated for the RVL strain together with the other rotavirus species reference strains indicate a distinct branching for RVL in the case of all analyzed amino acid sequences (Figure 1). With the exception of NSP4, RVL branches together with RVB, RVG, RVH, RVI and RVJ in one clade, whereas RVA, RVC, RVD and RVF form another clade. For NSP4, RVL clusters with RVA and RVG; however, the branching of this tree generally has a very low bootstrap support. 

## 4. Discussion

Rotaviruses comprise a large degree of sequence heterogeneity which is also reflected by their current classification into the 9 distinct species RVA to RVJ [14]. Two additional putative species RVK and RVL have been detected recently in common shrews [9], but they have been only scarcely characterized and their final classification is still pending. According to the ICTV regulations, a general determinant for grouping virus isolates into the same virus species within the family *Reoviridae* is their capacity to exchange genetic information by genome segment reassortment [13]. However, the reassortment capacity of rotaviruses cannot be easily determined by laboratory analysis so far. Therefore, additional criteria, such as the cross-reactivity of antibodies or amino acid sequence identity cut-off values have been defined for rotavirus species classification by the ICTV [13,15]. In our study, we determined the whole genome sequence of a putative RVL strain, thus providing an important prerequisite for further virus characterization and final classification. 

The genome of the RVL strain KS14/0241 has typical features of rotaviruses. This includes the presence of eleven genome segments, which encode proteins with similarities to the VPs and NSPs of other rotaviruses. For some proteins, only very low amino acid sequence identities were evident when compared to reference sequences of the other RV species. This was notably the case for NSP4; however, the observed low amino acid sequence identity for RVL was still within the range of sequence variability seen between other rotavirus species [16,26,27]. The VP4 and NSP3 of RVL are considerably larger than those of all other rotavirus species, and the function of the inserted amino acid sequences should be analyzed in future. In contrast, other proteins of RVL show higher similarities, e.g., VP1 with amino acid sequence identities up to 64% to that of RVJ, which most probably reflect highly conserved domains of this protein acting as RNA-dependent RNA polymerase [28,29]. The RVL major capsid protein VP6 has the highest amino acid sequence identity (47%) to that of RVH and RVJ. This is below the threshold of 53% used to differentiate members of different rotavirus species [15], thus supporting a grouping into a novel species RVL.

The non-coding 5′- and 3′-terminal sequences of the genome segments of RVL show conserved nucleotide positions. Those sequences are known to act as binding sites for VP1 and are required for virus-specific genome transcription and replication [30,31], as well as for NSP3, which may enhance translation [32]. A comparison with other rotavirus genomes indicates that the 5′-termini are conserved in all rotavirus species including RVL, with the consensus sequence GGN^A^/_U_ of the first four nucleotides. In contrast, the 3′-termini show three different consensus sequences used by the different rotavirus species, which is ACCC in the case of RVL. The same consensus sequence is present in the RVB-like clade (called clade B in [17] and clade 2 in [16]), consisting of RVB, RVG, RVH, RVI and RVJ. Structural modeling indicated that a different mechanism of template recognition is used by the VP1 molecules of this clade compared to that of the RVA-like clade [17]. 

The phylogenetic analysis showed separate branching of RVL for all encoded viral proteins, suggesting a distinct evolution of this virus, which resulted in a separate rotavirus species. Further analysis of the trees indicated a general grouping of the rotavirus species into the two phylogenetically distinct RVA-like and RVB-like clades, which are identical with the formerly designated clades A and B [17], or clades 1 and 2 [16], respectively. Originally, the RVA-like clade consisted of RVA, RVC, RVD and RVF, whereas the RVB-like clade contained RVB, RVG and RVH [16,17]. In recent years, the newly discovered rotavirus species RVI [26] and RVJ [27] have been added to the RVB-like clade and, according to our analysis, RVL has also to be grouped into this clade. The only tree, which does not reflect these clades is that based on NSP4. However, the very high sequence variation of NSP4 of rotaviruses [16,26,33], which is also reflected by very low bootstrap values in the corresponding tree, hinders the solid reconstruction of its evolutionary history so far.

Our study has some limitations. First of all, only one complete genome sequence was determined; therefore, the generation of additional genomic sequences would be desirable in the future in order to assess the genetic variability within the RVL species. Second, due to the used shrew trapping method we could not determine pathogenicity or virulence of the novel virus. As autolysis of organs of animals progresses rapidly, and that of the intestine in particular, it was impossible to look for pathological peculiarities or diarrhea using the applied approach. Further studies involving alive animals should be performed in order to determine the clinical importance of RVL. Third, the distribution of RVL in animals other than common shrews and its zoonotic potential for transmission to humans should be analyzed in future. Out of the RVB-like clade, at least representatives of RVB and RVH have been shown to infect both animals and humans, e.g., RVB has been detected in cattle, pigs and humans [2,3], and RVH in pigs and humans [3,34]. However, it is not known if distinct RVB or RVH strains have been adapted to specific host species, or if zoonotic transmission of the strains can occur. In further studies, samples of different animal species, in particular sympatric shrews and other small mammals at RVL-positive trapping sites, and of humans should be analyzed for the presence of RVL and detected strains should be characterized in order to determine the host range and zoonotic potential of RVL.

In conclusion, the generated data on amino acid sequence identities, conserved NCRs and phylogenetic reconstructions suggest a grouping of strain KS14/0241 into a new rotavirus species designated as RVL. Further sequence analyses indicate that RVL is related to the RVB-like class, which is also supported by phylogenetic data. Future research should focus on the distribution of the novel rotavirus species RVL in shrews and other hosts, its pathogenicity and its zoonotic potential for transmission to humans.

## Figures and Tables

**Table 1:** Deduced amino acid sequence identities of RVL strain KS14/0241 with the reference strains of other rotavirus species (as specified in Supplementary Data S1).

Gene Product	Amino Acid Sequence Identity (%)	
	Lowest (RV Species)	Highest (RV Species)
VP1	21 (RVA, RVD)	64 (RVJ)
VP2	12 (RVC, RVD, RVF)	63 (RVJ)
VP3	12 (RVC)	52 (RVH)
VP4	10 (RVD)	36 (RVJ)
NSP1 ^1^	6 (RVF)	22 (RVH)
VP6	10 (RVC)	47 (RVH, RVJ)
NSP3	8 (RVD)	29 (RVJ)
NSP2	12 (RVA, RVC)	49 (RVJ)
VP7	11 (RVC)	37 (RVJ)
NSP4	3 (RVF)	15 (RVA, RVJ)
NSP5	7 (RVF)	37 (RVJ)

^1^ for RVB, RVG and RVI: encoded amino acids of NSP1-2 [25] have been used.

**Table 2:** Comparison of the 5′- and 3′-terminal (positive-sense RNA strand) genome segment sequences of RVL strain KS14/0241 with the reference strain sequences of the other rotavirus species (as specified in Supplementary Data S1). Nucleotides present at the same position of the NCRs in all segments of RVL and consensus sequences of all rotaviruses belonging to a clade (RVA-, RVB-like) or RVC are shown in bold.

Gene Encoded by the Segment or Rotavirus Species and Clade	5′-Terminus	3′-Terminus
***RVL segments***		
VP1	**GGC**ACGAUGGAUGAA	UAGCAC**A**A**UAGACCC**
VP2	**GGC**ACUUAGCUUAGA	AGACUU**A**A**UAGACCC**
VP3	**GGC**AUAUGAUGUCGA	CAAUAG**A**A**UAGACCC**
VP4	**GGC**ACUUAGCAAGAU	AGCUAC**A**G**UAGACCC**
VP6	**GGC**UCGACAAGGUUA	AUAUAC**A**A**UAGACCC**
VP7	**GGC**ACAUUCAAAUUC	UUAGCA**A**A**UAGACCC**
NSP1	**GGC**UCAUUUAAGUUC	AAAUAC**A**C**UAGACCC**
NSP2	**GGC**UCUUCACCAUAG	ACAAAU**A**U**UAGACCC**
NSP3	**GGC**ACAGAUAGUUAG	CUACAC**A**U**UAGACCC**
NSP4	**GGC**ACCCUUAGCAUC	AGCACA**A**U**UAGACCC**
NSP5	**GGC**UCAUUUGAAGAG	GGAAUG**A**A**UAGACCC**
***Genome consensus***		
RVL	GGCAU	UAGACCC
RVB	GGCUA	AAAACCC
RVG	GGCAA	AAGACCC
RVH	GGAUA	UAUACCC
RVI	GGCUA	AAAACCC
RVJ	GGCAA	UANACCC
**RVB-like clade**	GGNAU	**ACCC**
		
RVA	GGCU	UGUGACC
RVD	GGCUAU	UGUGACC
RVF	GGCAU	UNUGACC
**RVA-like clade**	GGNAU	**GACC**
		
RVC	GGCAU	UGUGGCU
**RVC**	GGCAU	**GGCU**

## Data Availability

Data are available as Supplementary Data S1–S4 and additional data can be retrieved upon request from R.J. The GenBank accession numbers of the nucleotide sequences of the shrew rotavirus identified in this study are presented in Section 2.3.

## Supplementary Materials

The following supporting information can be downloaded at: https://www.mdpi.com/article/10.3390/v14030462/s1, Supplementary Data S1: Rotavirus strains and GenBank accession numbers of sequences used for sequence comparisons and phylogenetic trees. Supplementary Data S2: Sizes of genomes, genome segments and encoded proteins of rotavirus L compared to other rotavirus species. Supplementary Data S3: Alignment of deduced amino acid sequences of VP4 of rotavirus reference sequences of rotavirus species A to L. Supplementary Data S4: Alignment of deduced amino acid sequences of NSP3 of rotavirus reference sequences of rotavirus species A to L.
