# Supplementary material for: Whole Genome Sequence Analysis of a Prototype Strain of the Novel Putative Rotavirus Species L
Source: Viruses. 2022 Feb 24;14(3):462. doi: 10.3390/v14030462 (PMC8954357; doi:10.3390/v14030462)

Supplementary Data for

"Whole Genome Sequence Analysis of a Prototype Strain of the Novel Putative Rotavirus Species L"

Reimar Johne, Katja Schilling-Loeffler, Rainer G. Ulrich and Simon H. Tausch

Supplementary Data S1: Rotavirus strains and GenBank accession numbers of sequences used for sequence comparisons and phylogenetic trees.

***Rotavirus species*: strain**	**Encoded protein**	**GenBank acc.-no.**
Rotavirus A: RVA/Simian-tc/ZAF/SA11-H96/1958/G3P5B[2]	VP1	X16830
	VP2	X16831
	VP3	X16062
	VP4	X14204
	VP6	X00421
	VP7	K02028
	NSP1	X14914
	NSP2	J02353
	NSP3	X00355
	NSP4	KO1138
	NSP5	X07831
Rotavirus B: RVB/Human-wt/BGD/Bang373/2000/GXP[X]	VP1	EU490415
	VP2	AY238390
	VP3	EU490418
	VP4	AY238388
	VP6	AY238389
	VP7	AY238385
	NSP1	AY238391
	NSP2	AY238393
	NSP3	AY238392
	NSP4	AY238384
	NSP5	AY238394
Rotavirus C: RVC/Human-tc/GBR/Bristol/1988/G4P[2]	VP1	AJ304859
	VP2	AJ303139
	VP3	X96697
	VP4	X79442
	VP6	X59843
	VP7	X77257
	NSP1	AJ132204
	NSP2	AJ132205
	NSP3	AJ132203
	NSP4	X83967
	NSP5	M81488
Rotavirus D: RVD/Chicken-wt/DEU/05V0049/2005/GXP[X]	VP1	GU733443
	VP2	GU733444
	VP3	GU733446
	VP4	GU733445
	VP6	GU733448
	VP7	GU733451
	NSP1	GU733447
	NSP2	GU733450
	NSP3	GU733449
	NSP4	GU733452
	NSP5	GU733453

Supplementary Data S1 (cont.)

Rotavirus F: RVF/Chicken-wt/DEU/03V0568/2003/GXP[X]	VP1	JN596591
	VP2	JQ919995
	VP3	JQ919996
	VP4	JQ919997
	VP6	HQ403603
	VP7	JQ919998
	NSP1	JQ919999
	NSP2	JQ920000
	NSP3	JQ920001
	NSP4	JQ920002
	NSP5	JQ920003
Rotavirus G: RVG/Chicken-wt/DEU/03V0567/2003/GXP[X]	VP1	JN596592
	VP2	JQ920004
	VP3	JQ920005
	VP4	JQ920006
	VP6	HQ403604
	VP7	JQ920007
	NSP1	JQ920008
	NSP2	JQ920009
	NSP3	JQ920010
	NSP4	JQ920011
	NSP5	JQ920012
Rotavirus H: RVH/Human-tc/CHN/NADRV-J19/1997/GXP[X]	VP1	DQ113897
	VP2	DQ113898
	VP3	DQ113900
	VP4	DQ113899
	VP6	DQ113902
	VP7	DQ113905
	NSP1	DQ113901
	NSP2	DQ113903
	NSP3	DQ113904
	NSP4	DQ113906
	NSP5	DQ113907
Rotavirus I: RVI/Dog-wt/HUN/KE135/2012/GXP[X]	VP1	KM369892
	VP2	KM369893
	VP3	KM369894
	VP4	KM369895
	VP6	KM369896
	VP7	KM369897
	NSP1	KM369887
	NSP2	KM369888
	NSP3	KM369889
	NSP4	KM369890
	NSP5	KM369891

Supplementary Data S1 (cont.)

Rotavirus J: RVJ/Bat-wt/SRB/BO4351/Ms/2014 GXP[X]	VP1	KX756624
	VP2	KX756625
	VP3	KX756626
	VP4	KX756627
	VP6	KX756628
	VP7	KX756629
	NSP1	KX756619
	NSP2	KX756620
	NSP3	KX756621
	NSP4	KX756622
	NSP5	KX756623
***Rotavirus L*: RVL/shrew-wt/GER/KS14-0241/2013/GXP[X]**	**VP1**	**OM101015**
	**VP2**	**OM101016**
	**VP3**	**OM101018**
	**VP4**	**OM101017**
	**VP6**	**OM101020**
	**VP7**	**OM101023**
	**NSP1**	**OM101021**
	**NSP2**	**OM101022**
	**NSP3**	**OM101019**
	**NSP4**	**OM101024**
	**NSP5**	**OM101025**

Supplementary Data S2: Sizes of genomes, genome segments and encoded proteins of rotavirus L compared to other rotavirus species.

**Encoded protein**	**Size of genome segment or genome in nucleotide numbers (encoded amino acid numbers)**									
	**RVA**	**RVB**	**RVC**	**RVD**	**RVF**	**RVG**	**RVH**	**RVI**	**RVJ**	**RVL**
**VP1**	3302 (1088)	3511 (1160)	3309 (1090)	3274 (1080)	3296 (1086)	3526 (1160)	3538 (1167)	3518 (1162)	3533 (1168)	**3543 (1168)**
**VP2**	2690 (881)	2847 (934)	2736 (884)	2801 (914)	2769 (904)	3014 (991)	2969 (973)	3002 (983)	3010 (986)	**2981 (981)**
**VP3**	2591 (835)	2341 (764)	2166 (693)	2104 (686)	2174 (694)	2352 (768)	2204 (719)	2161 (701)	2200 (715)	**2192 (715)**
**VP4**	2362 (776)	2306 (750)	2283 (744)	2366 (778)	2246 (738)	2364 (772)	2512 (823)	2371 (777)	2512 (826)	**2598 (852)**
**NSP1***	1611 (495)	1276 (321)	1270 (394)	1872 (574)	1791 (547)	1295 (324)	1307 (395)	1485 (390)	1322 (401)	**1052 (326)**
**VP6**	1356 (397)	1269 (391)	1353 (395)	1353 (399)	1314 (396)	1267 (391)	1287 (396)	1278 (395)	1277 (395)	**1274 (396)**
**NSP3**	1104 (315)	1179 (347)	1350 (402)	1242 (371)	1309 (370)	1052 (303)	932 (262)	954 (273)	1108 (331)	**1405 (425)**
**NSP2**	1059 (317)	1007 (301)	1037 (312)	1026 (311)	1068 (318)	1012 (282)	1004 (297)	1018 (301)	1017 (299)	**1037 (310)**
**VP7**	1062 (326)	814 (249)	1063 (332)	1025 (317)	990 (295)	825 (247)	820 (258)	858 (270)	793 (245)	**814 (245)**
**NSP4**	751 (175)	751 (219)	613 (150)	765 (128)	678 (169)	801 (187)	739 (213)	751 (219)	743 (210)	**720 (204)**
**NSP5**	667 (198)	631 (170)	730 (212)	672 (195)	706 (218)	678 (181)	649 (176)	593 (157)	620 (165)	**684 (186)**
**Genome**	18,555	17,932	17,910	18,500	18,341	18,186	17,961	17,989	18,135	**18,300**

* for RVB, RVG and RVI: encoded amino acids of NSP1-2 (Diller et al., J. Virol. 2019, 93, e00813-19) are shown.

Supplementary Data S3: Alignment of deduced amino acid sequences of VP4 of rotavirus reference sequences of rotavirus species A (top sequence) to L (bottom sequence). Amino acid residues with similar chemical properties are shown in the same colors. A consensus sequence is shown above the alignment and sequence logos of the amino acid positions below the alignment. The alignment was performed using the MegAlign Pro module of the DNASTAR software package (Lasergene, Madison, WI, USA) by the ClustalW method with default parameters.

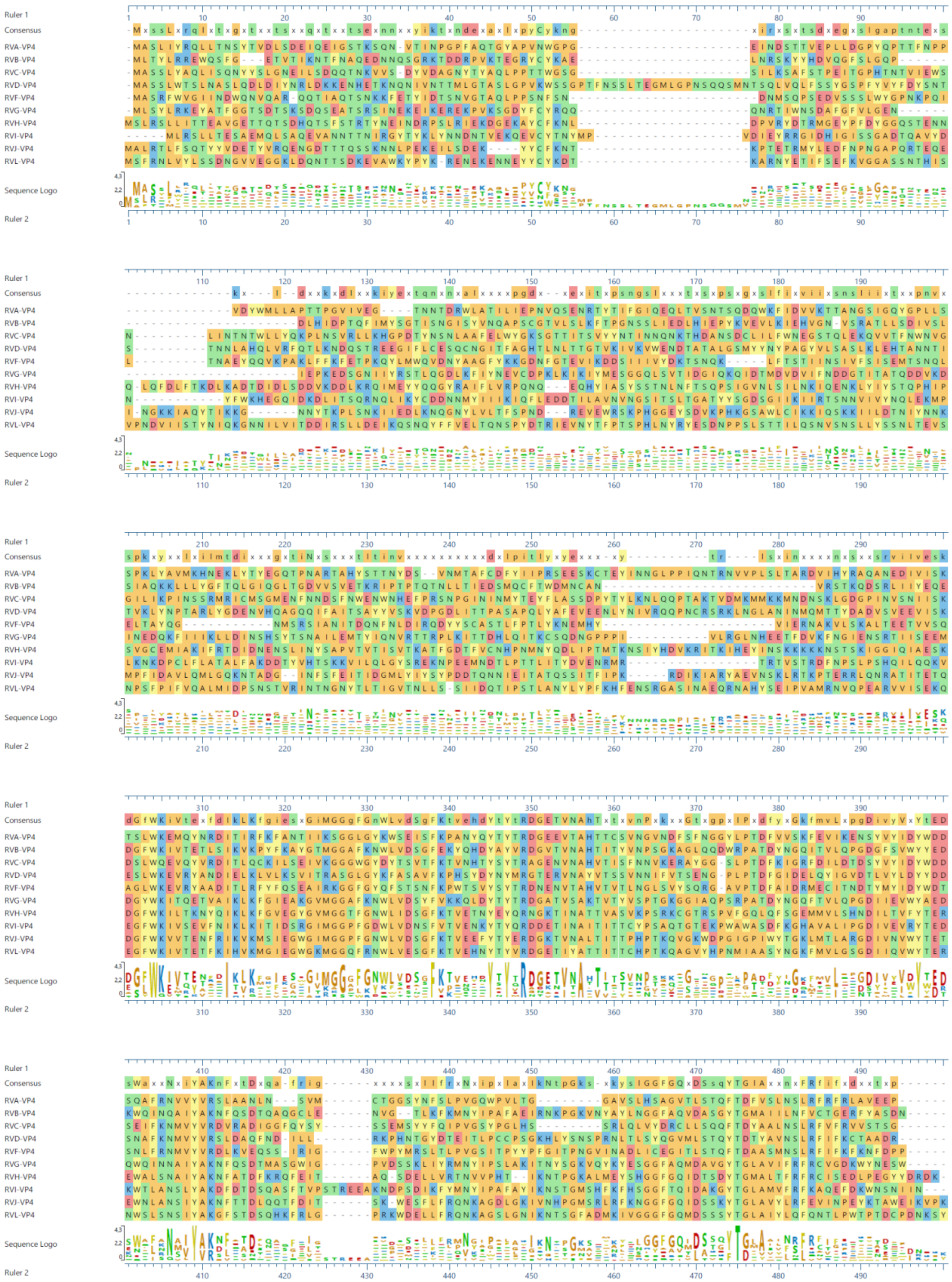

Supplementary Data S3 (cont.)

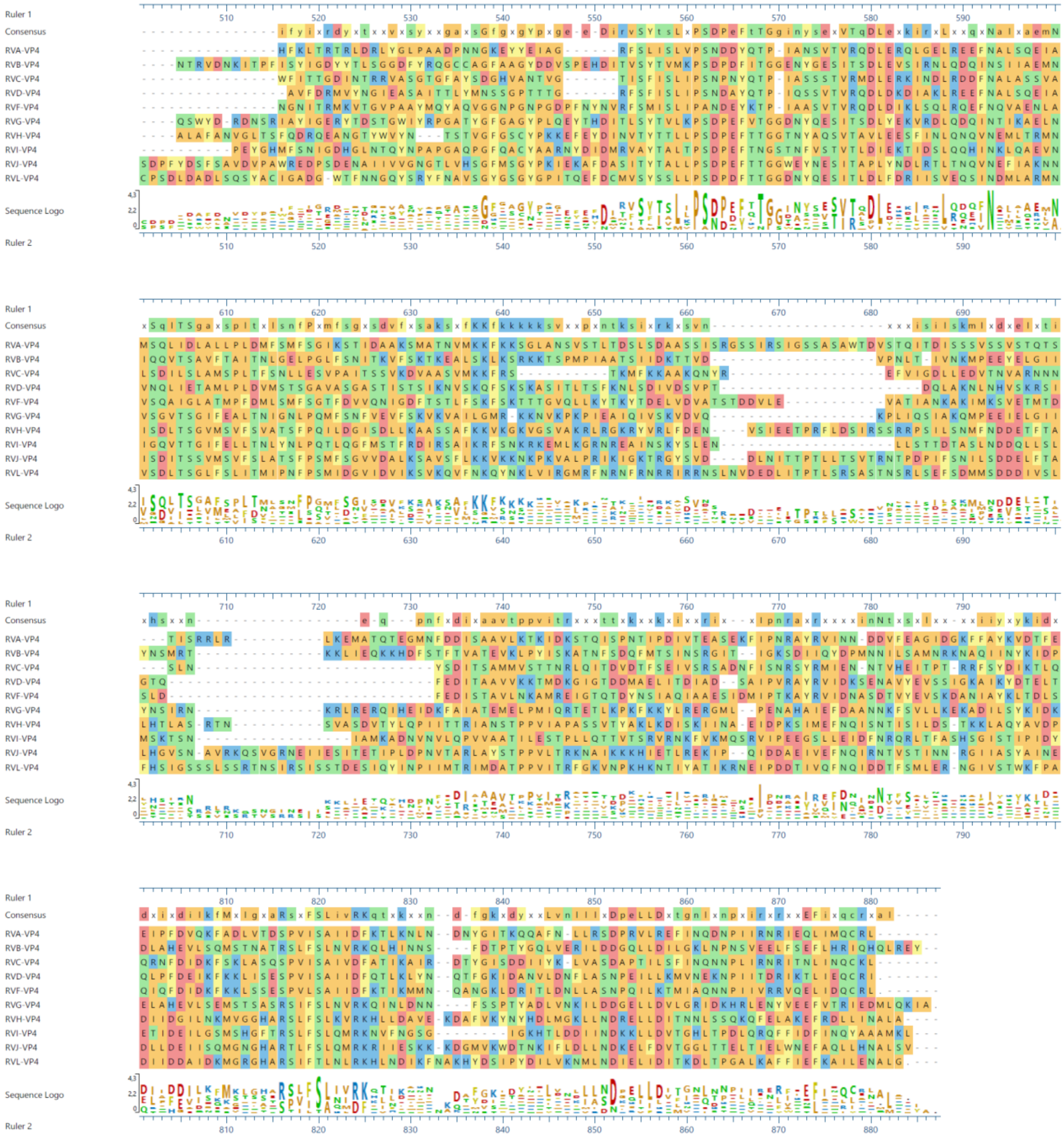

Supplementary Data S4: Alignment of deduced amino acid sequences of NSP3 of rotavirus reference sequences of rotavirus species A (top sequence) to L (bottom sequence). Amino acid residues with similar chemical properties are shown in the same colors. A consensus sequence is shown above the alignment and sequence logos of the amino acid positions below the alignment. The alignment was performed using the MegAlign Pro module of the DNASTAR software package (Lasergene, Madison, WI, USA) by the ClustalW method with default parameters.

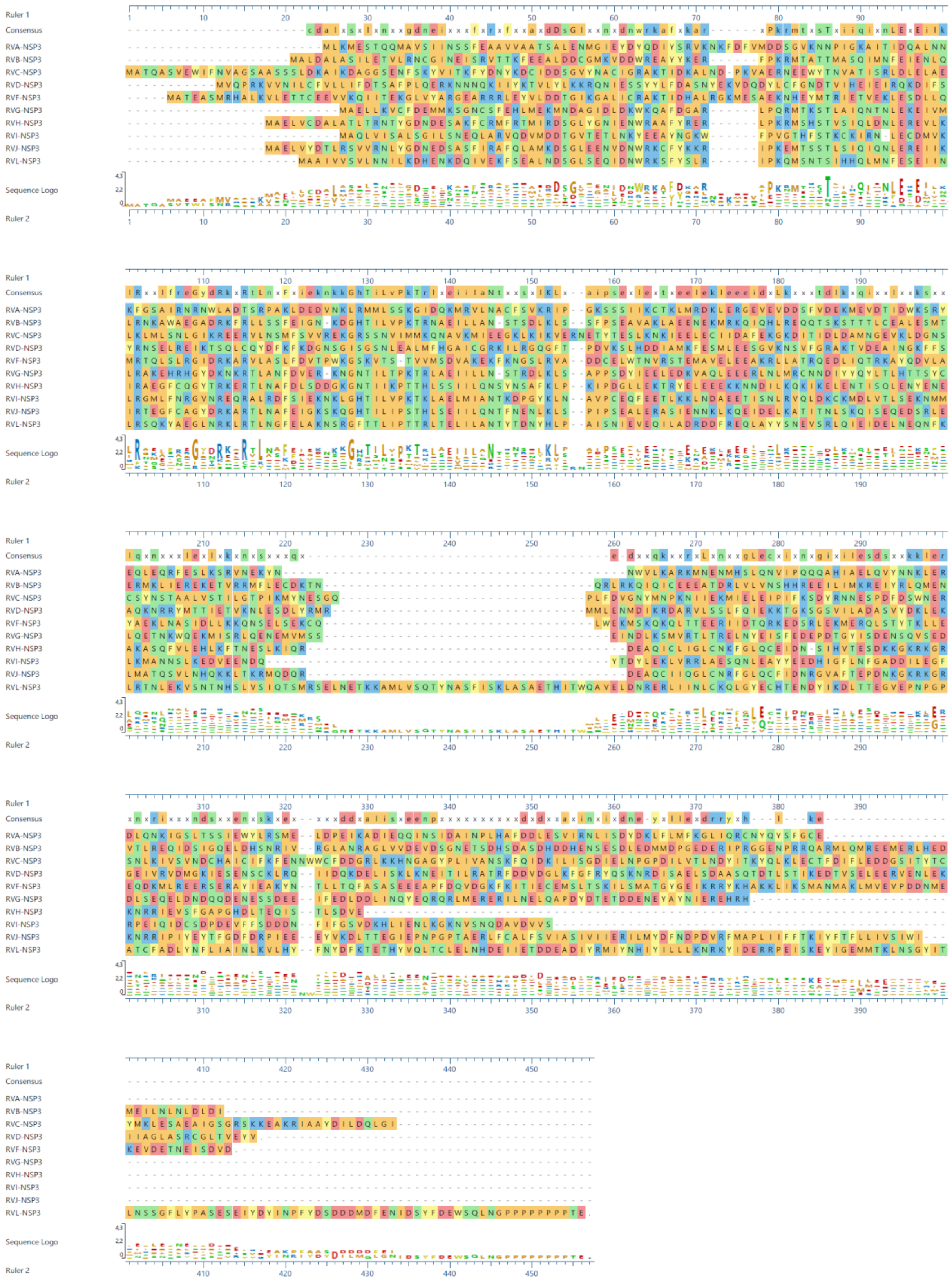

Supplement: Supplementary file 1 [file viruses-14-00462-s001.zip › viruses-1608724-supplementary.pdf]
